# Allogeneic hematopoietic stem cell transplantation in non‐Hodgkin lymphoma in Switzerland, 30 years of experience: Sooner is better

**DOI:** 10.1002/jha2.614

**Published:** 2022-11-13

**Authors:** Ekaterina Rebmann, Mitja Nabergoj, Bastien Grandjean, Paraskevi Stakia, Alix Stern, Michael Medinger, Stavroula Masouridi‐Levrat, Carole Dantin, Urs Schanz, Helen Baldomero, Jakob Passweg, Gayathri Nair, Alicia Rovo, Yves Chalandon

**Affiliations:** ^1^ Department of Hematology University Hospital of Bern (Inselspital) Bern Switzerland; ^2^ Department of Hematology L'Hôpital Riviera‐Chablais Vaud‐Valais Switzerland; ^3^ Department of Hematology Geneva University Hospitals (HUG) Genève Switzerland; ^4^ Department of Oncology‐Hematology Hospital of Neuchâtel (RHNE) Neuchâtel Switzerland; ^5^ Department of Hematology University Hospital of Basel (USB) Basel Switzerland; ^6^ Department of Hematology University Hospital of Zurich (UZH) Zürich Switzerland; ^7^ SBST Data Registry Office Univesiry Hospital of Basel Basel Switzerland; ^8^ Faculty of Medicine University of Basel Basel Switzerland; ^9^ Faculty of Medicine University of Geneva Geneva Switzerland

**Keywords:** GVHD, lymphomas, stem cell transplantation

## Abstract

Due to relatively high nonrelapse mortality (NRM), allogeneic hematopoietic stem cell transplantation (allo‐HSCT) in non‐Hodgkin's lymphoma (NHL) remains the ultimate line of treatment but the only curable approach in a setting of relapse/refractory disease. Here, we conducted a retrospective, multicenter, registry‐based analysis on patients who underwent allo‐HSCT for NHL in Switzerland, over 30‐year (1985–2020) period. The study included 301 allo‐HSCTs performed for NHL patients in three University Hospitals of Switzerland (Zurich, Basel and Geneva) 09/1985 to 05/2020. We assessed in univariate and multivariable analysis the impact on survivals (overall survival [OS], relapse free survival [RFS], relapse incidence [RI], and non‐treatment related mortality [NRM]). The maximum follow‐up was 25 years with median follow‐up for alive patients of 61 months. The median age at allo‐HSCT was 51 years. Three‐ and ‐year OS was ‐ 59.5% and 55.4%; 3‐ and 5‐year PFS was 50% and 44%; 3‐ and 5‐year NRM was 21.7% and 23.6%. RI at 3 and 5 years was 27.4% and 34.9%. In conclusion, our analysis of the entire Swiss experience of allo‐HSCT in patients with NHL shows promising 5‐ and possibly 10‐year OS and relatively acceptable NRM rates for such population, the majority being not in complete remission (CR) at the time of transplantation.

## 1 INTRODUCTION

Due to relatively high nonrelapse mortality (NRM), allogeneic hematopoietic stem cell transplantation (allo‐HSCT) in non‐Hodgkin's lymphoma (NHL) remains the ultimate/first choice line of treatment.

However, despite rapid development of new highly efficient targeted and cellular‐therapies, allo‐HSCT currently represents the only potentially curable treatment for virtually all type of NHL in a setting of relapse/refractory disease (rrNHL)[1, 2, 3].

The introduction of reduced intensity conditioning regimens (RIC), usage of T‐depletion methods, amelioration of supportive care and graft‐versus–host disease (GvHD) prevention, and treatment have reduced the NRM rates, thus improving allo‐HSCT outcomes and decreasing the gap with the one of autologous HSCT (auto‐HSCT) in NHL [4, 5, 6, 7, 8, 9, 10].

## 2 MATERIALS AND METHODS

Here, we conducted a retrospective, multicenter, registry‐based analysis on patients who underwent an allo‐HSCT for NHL in Switzerland, over a 30‐year (1985–2020) period. The study included 301 allo‐HSCTs performed for NHL patients in three University Hospitals of Switzerland (Zurich, Basel and Geneva) between September 1985 and May 2020; the data were reported to the Swiss Blood Stem cell Transplantation group (SBST). All patients whose transplant data are recorded in SBST by participating centers provide informed consent authorizing the use of their personal information for research purposes according to the ethical principles of the Declaration of Helsinki.

The NHL cohort included chronic lymphocytic leukemia (CLL) = 53, diffuse large B‐cell lymphoma (DLBCL) = 76, mantle cell lymphoma (MCL) = 33, follicular lymphoma (FL) = 30, marginal zone lymphoma (MZL) = 8, Burkitt lymphoma (BL) = 10, Waldenström macroglobulinemia (WM) = 6, hairy cell leukemia (HCL) = 1, peripheral T‐cell, not otherwise specified (T‐NOS) = 54, anaplastic large cell lymphoma (ALCL) = 7 and angioimmunoblastic T‐cell lymphoma (AITL) = 23. Further clinical characteristics are presented in Table 1.

**TABLE 1:** Patient's clinical characteristics

Parameters	Data	Missing
Age at allo‐HSCT, years (median, IQR)	51 (43–58)	
<50 years, *n* (%)	131.(44)	
≥50 years, *n* (%)	170 (56)	
Age at NHL diagnosis, years (median, IQR)	48 (39–54)	
Year of alloSCT (median, IQR)	2012 (2006–2016)	
Before 2000, *n* (%)	24 (9)	
After 2000, *n* (%)	277 (91)	
Prior autoSCT, *n* (%)	130 (43)	
Sex, *n* (%)		0
Male	206 (68)	
Female	95 (32)	
Disease type, *n* (%)		0
T	84 (28)	
B	217 (72)	
NHL subtype, *n* (%)		0
CLL	53 (18)	
DLBCL	76 (25)	
MCL	33 (11)	
FL	30 (10)	
MZL	8 (3)	
BL	10 (3)	
T cell others	54 (18)	
ALCL	7 (2)	
AITL	23 (7)	
WM	6 (2)	
HCL	1 (1)	
Grading, *n* (%)		0
Indolent	156 (52)	
Aggressive	145 (48)	
Karnofsky, *n* (%)		14
<80	28 (10)	
≥80	259 (90)	
Disease status at alloSCT, *n* (%)		0
CR	144 (48)	
PR	73 (24)	
SD	13 (4)	
PD	56 (19)	
Primary refractory	7 (2)	
Unknown	8 (3)	
Number_times_response_achieved, *n* (%)		185
0	40 (34)	
1	76 (66)	
Stem cell source, *n* (%)		0
BMSC	261 (87)	
PBSC	40 (13)	
Ex‐vivo manipulation, *n* (%)		55
No	210 (85)	
Yes	36 (15)	
Type of donor, *n* (%)		0
HLA‐identical sibling	159 (53)	
HLA‐matched unrelated donor	74 (5)	
MMUD	3 (1)	
PMRD	25 (8)	
Syn	8 (3)	
UD‐NOS	32 (11)	
TBI, *n* (%)		0
Yes	132 (44)	
No	169 (56)	
Conditioning regimen, *n* (%)		15
MAC	119 (42)	
RIC	167 (58)	
HLA mismatch, *n* (%)		167
0	126 (94)	
1	1 (1)	
≥2	7 (5)	
ABO matching, *n* (%)		240
Compatible	35 (57)	
Major incompatibility	15 (25)	
Minor incompatibility	11 (18)	
Donor age, years (median, IQR)	41 (30–52)	
Engraftment, *n* (%)		22
Yes	273 (98)	
No	6 (2)	
Neutrophil engraftment, days (median, IQR)	14 (12–18)	

Abbreviations: AITL, angioimmunoblastic T‐cell lymphoma; ALCL, anaplastic large cell lymphoma; allo‐HSCT, allogeneic hematopoietic stem cell transplantation; BMSC, bone marrow‐derived stem cell; BL, Burkitt lymphoma; CLL, chronic lymphocytic leukaemia; CR, complete remission; DLBCL, diffuse large B‐cell lymphoma; FL, follicular lymphoma; HCL, hairy cell leukemia; HLA, human leukocyte antigen; IGR, interquartile range; MAC, myeloablative conditioning; MCL, mantle cell lymphoma; MMUD, mismatched unrelated donor; MZL, marginal zone lymphoma; NHL, non‐Hodgkin's lymphoma; PBSC, peripheral blood‐derived stem cell; PD, progressive disease; PMRD, partially mismatched related donor; PR, partial remission; RIC, reduced intensity conditioning; SD, stable disease; TBI, total body radiation; T‐NOS, T‐cell, not otherwise specified; UD, Unrelated donor; WM, Waldenström macroglobulinemia.

We assessed in univariate and multivariable analysis the impact on survivals (overall survival [OS], relapse free survival [RFS], relapse incidence [RI] and NRM).

## 3 RESULTS

The maximum observed follow‐up was 25 years with a median follow‐up for alive patients of 61 months (interquartile range: 24–112). The median age for patients at the time of allo‐HSCT was 51 years (IQR 43–58). The 3‐ and 5‐year OS was 59.5% (standard error ± 5.0%) and 55.4% (± 5.6%), respectively, 3‐ and 5‐year PFS was 50.0% (±6.1%) and 44.1% (±7.1%), respectively, and 3‐ and 5‐year NRM 21.7% (±2.5%) and 23.6% (±2.7%), respectively. RI at 3 and 5 years was 27.4% (±2.7%) and 34.9% (±3.2%), respectively.

According to the univariate analysis for OS and RFS, patients transplanted at a younger age (<50 years) with a good performance status (Karnofsky index ≥80), those in CR, especially in the first one (vs. second CR or above) and those transplanted with a human leukocyte antigen (HLA)‐matched sibling showed significantly better outcomes (*p* < 0.05). Total body radiation (TBI) use was also related with a trend toward better OS and RFS (*p* = 0.058 and 0.06, respectively). Of note, a survival plateau was observed after 5 years of follow‐up. T‐cell histology was associated with a higher relapse risk, as did a poor performance status and the exclusion of TBI in the conditioning regimen.

**FIGURE 1:**
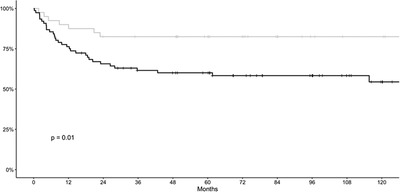
Overall survival (OS) if transplanted in first remission

An important message from our retrospective study was the surprisingly good OS in NHL patients, transplanted in first complete remission (Figure 1). Significantly, this subgroup included NHL with known dismal prognosis, as AITL, MCL, and T‐NHL NOS (Table 2). As it was already shown, the late usage of allo‐HSCT decreases its efficacy, and the number of prior chemotherapy regimens negatively influences the global outcome of allo‐HSCT [5].

**TABLE 2:** Basic clinic characteristics of patients transplanted in first CR

**NHL type**	**Patient number**	**Median age**	**Alive**	**Median follow‐up (months)**
CLL	7 (17.5%)	53	6/7 (90%)	86
DLBCL	11 (27.5%)	49	10/11 (90%)	60
MCL	6 (15%)	51	5/6 (90%)	90
FL	2 (5%)	42	1/2 (50%)	50
BL	1(2.5%)	36	Alive	83
T‐cell, NOS	7 (17.5%)	39	5/7 (70%)	55
ALCL	3 (7.5%)	29	2/3 (90%)	7.5
AITL	3 (7.5%)	37	3 (100%)	162
**Total**	***N* = 40**	**42**	**83%**	**74**

Abbreviations: AITL, angioimmunoblastic T‐cell lymphoma; ALCL, anaplastic large cell lymphoma; BL, Burkitt lymphoma; CLL, chronic lymphocytic leukaemia; DLBCL, diffuse large B‐cell lymphoma; FL, follicular lymphoma; NHL, non‐Hodgkin's lymphoma; MCL, mantle cell lymphoma; T‐NOS, T‐cell, not otherwise specified.

To date, the absence of prospective randomized trials focused on allogeneic transplantation in NHL, leaves unclearthe best timing for its usage  [11, 12].

## 4 CONCLUSIONS

In conclusion, our analysis of the entire Swiss experience/knowledge/research of allo‐HSCT in patients with NHL shows promising 5‐ and possibly 10‐year OS and relatively acceptable NRM rates for such a heavily pretreated, the majority being not in CR at time of transplantation.

The present analysis supports the idea that an earlier usage of allo‐HSCT in settings of rrNHL might contribute to significant improvement of survival rather than in the latter lines/later stages. Although validating these data prospectively would be advisable, we recognize that this would not be feasible due to the development of new treatment modalities. Nevertheless, these data could serve as a comparator/comparison for new therapies, particularly cellular therapies such as chimeric antigen receptor T‐cells [1, 13–16].

## AUTHOR CONTRIBUTIONS

*Data collection, analysis, interpretation, and manuscript writing*: E.R. *Data analysis and statistic*: M.N. *Data collection and interpretation*: B.G. *Data collection and interpretation*: P.S. *Data collection and analysis*: H.B., M.M., S.M.‐L., C.D., N.G, A.S., U.S., J.P., and A.R. *Data interpretation and manuscript*: Y.C.

## CONFLICT OF INTEREST

The authors have no conflict of interest to declare that is relevant to the content of this article.

## FUNDING INFORMATION

The authors did not receive support from any organization for the submitted work.

## ETHICS STATEMENT

All procedures were in accordance with the ethical standards of the respective local research committee and with the 1964 Helsinki declaration and its later amendments or comparable ethical standards. Informed written consent was obtained from all individual participants included in the SBST register.

## Data Availability

The data that support the findings of this study are available upon request from the corresponding author. The data are not publicly available due to privacy or ethical restrictions.
